# Effect of pirfenidone in patients with more advanced idiopathic pulmonary fibrosis

**DOI:** 10.1186/s12931-019-1021-2

**Published:** 2019-03-12

**Authors:** Ulrich Costabel, Carlo Albera, Marilyn K. Glassberg, Lisa H. Lancaster, Wim A. Wuyts, Ute Petzinger, Frank Gilberg, Klaus-Uwe Kirchgaessler, Paul W. Noble

**Affiliations:** 1 Ruhrlandklinik, University Hospital, University of Duisburg-Essen, Tüschener Weg, 45239 Essen, Germany; 2 Department of Clinical and Biological Sciences, Interstitial and Rare Lung Disease Unit, University of Turin, Regione Gonzole 10, 10043 Orbassano, Italy; 3 Departments of Medicine, Surgery, and Pediatrics, University of Miami Health System, 1321 14th Street, Suite 510, Miami, FL 33125 USA; 4 Department of Medicine, Vanderbilt University Medical Center, 1211 Medical Center Drive, Nashville, TN 37232 USA; 5 Department of Respiratory Medicine, Unit for Interstitial Lung Diseases, University Hospitals Leuven, Herestraat 49, 3000 Leuven, Belgium; 6 Accovion GmbH, Helfmann-Park 10, 65760, Eschborn, Germany; 7 F. Hoffmann-La Roche, Ltd., Konzem-Hauptsitz, Grenzacherstrasse 124, CH-4070 Basel, Switzerland; 8 Department of Medicine, Cedars-Sinai Medical Center, 8700 Beverly Blvd, Los Angeles, CA 90048 USA

**Keywords:** Advanced disease, Antifibrotic, Idiopathic pulmonary fibrosis, Lung function, Pirfenidone

## Abstract

Data from controlled clinical studies in patients with more advanced idiopathic pulmonary fibrosis (IPF) could inform clinical practice, but they are limited, since this sub-population is usually excluded from clinical trials. These exploratory post-hoc analyses of the open-label, long-term extension study RECAP (NCT00662038) aimed to assess the efficacy and safety of pirfenidone in patients with more advanced IPF. Patients were categorised according to the extent of lung function impairment at baseline: more advanced (percent predicted FVC <50% and/or DLco <35%) and less advanced (percent predicted FVC ≥50% and DLco ≥35%).

Overall, 596 patients with baseline FVC and/or DLco values available were included in the analyses; 187 patients had more advanced disease, and 409 patients had less advanced disease. Mean percent predicted FVC declined throughout 180 weeks of treatment in both more and less advanced disease subgroups. Both subgroups exhibited a similar pattern of adverse events; however, adverse events related to IPF progression were experienced by a higher proportion of patients with more advanced versus less advanced disease. Discontinuation rates due to any reason, adverse events related to IPF progression, or deaths were each higher in the more advanced versus the less advanced disease subgroup.

These analyses found that longer-term pirfenidone treatment resulted in a similar rate of lung function decline and safety profile in patients with more advanced versus less advanced IPF, and the data suggest that pirfenidone is efficacious, well tolerated, and a feasible treatment option in patients with more advanced IPF.

## Background

Idiopathic pulmonary fibrosis (IPF) is a debilitating, progressive, fatal, fibrosing lung disease [1, 2]. Lung function, measured by percent predicted forced vital capacity (FVC) or carbon monoxide diffusing capacity (DLco), correlates with IPF disease outcomes, with more advanced impairments associated with decreased health-related quality of life [3] and survival [4]. Data from controlled clinical studies in patients with more advanced disease, which could inform clinical practice, are limited, since this subpopulation is usually excluded from clinical trials in IPF [5–8]. For example, in the phase III ASCEND (NCT01366209) and CAPACITY trials (NCT00287729 and NCT00287716) of pirfenidone in patients with IPF, patients with percent predicted FVC <50%, or DLco <30% (ASCEND) or <35% (CAPACITY), were excluded [6, 7]. Nevertheless, in daily practice, patients would be expected to remain on treatment if their percent predicted FVC or DLco decreased to <50% or <35%, respectively. These exploratory post-hoc analyses of RECAP aimed to assess efficacy and safety of pirfenidone in patients with more advanced IPF.

## Methods

RECAP (PIPF-012; NCT00662038) was an open-label, long-term extension study in patients with IPF who had completed ASCEND or CAPACITY (there were no restrictions on disease severity for entry into RECAP), the methods and primary outcomes of which have been described previously [9]. Patients who previously received pirfenidone or placebo in CAPACITY and received pirfenidone 2403 mg/day during RECAP were included in the analyses. Patients from ASCEND were not included due to lack of FVC follow-up data [9].

Patients were categorised according to IPF severity, assessed by lung function impairment at baseline (entry into RECAP): more advanced (percent predicted FVC < 50% and/or DLco <35%) and less advanced (percent predicted FVC ≥50% and DLco ≥35%; FVC ≥50% or DLco ≥35%, if other lung function data were missing). Efficacy of pirfenidone by IPF severity was assessed by decline in percent predicted FVC and FVC volume over 180 weeks, as measured using change from baseline and linear slope analysis of annual rate of decline. Safety of pirfenidone by IPF severity was assessed by adverse event (AE) occurrence and reasons for discontinuation over 180 weeks.

## Results

Overall, 628/779 (80.6%) patients completed CAPACITY without discontinuing treatment [6]; of these, 603 patients were enrolled in RECAP (*n =* 68 and *n =* 261 had received previous pirfenidone treatment at 1197 and 2403 mg/day, respectively; *n =* 274 had previously received placebo). In total, 596 patients with baseline FVC and/or DLco values available were included in these analyses; 187 patients had more advanced disease (*n =* 100, previous pirfenidone group; *n =* 87, previous placebo group) and 409 patients had less advanced disease (*n =* 225, previous pirfenidone group; *n =* 184, previous placebo group). Demographics in both the previous pirfenidone and placebo groups, respectively, were similar for more versus less advanced disease subgroups (mean age, 68.1 years and 68.0 years vs 68.3 years and 68.4 years; male, 72.0 and 78.2% vs 70.7 and 70.7%; white, 98.0 and 100.0% vs 97.8 and 96.7%; mean body mass index, 28.9 kg/m^2^ and 30.0 kg/m^2^ vs 29.2 kg/m^2^ and 29.8 kg/m^2^). Mean (standard deviation) percent predicted FVC at baseline for the previous pirfenidone and placebo groups, respectively, was 61.0% (14.3) and 58.4% (13.7) for more advanced disease, and 76.0% (15.2) and 76.1% (15.4) for less advanced disease. Corresponding values for percent predicted DLco were 29.5% (5.9) and 28.8% (6.2), respectively, for more advanced disease, and 46.7% (11.4) and 47.4% (8.8), respectively, for less advanced disease. Mean duration of pirfenidone exposure over 180 weeks of treatment was 102.3 weeks for the more advanced and 138.1 weeks for the less advanced disease subgroup.

**Fig. 1:**
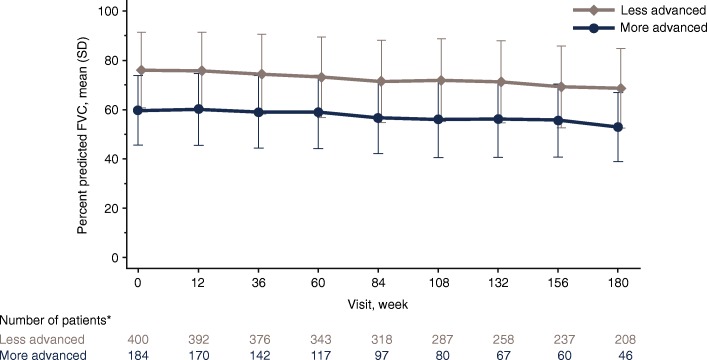
Mean percent predicted FVC over time by IPF severity at baseline in RECAP. *Patients with missing percent predicted FVC and DLco values were excluded. *FVC* Forced vital capacity, *DLco* Carbon monoxide diffusing capacity, *IPF* Idiopathic pulmonary fibrosis, *SD* Standard deviation

Mean percent predicted FVC declined throughout 180 weeks of treatment in both more and less advanced disease subgroups (Fig. 1). In the more advanced disease subgroup, mean (standard error) annual rate of percent predicted FVC decline was 3.8% (0.40) and 3.4% (0.43) following previous pirfenidone and placebo treatment, respectively. Corresponding values in the less advanced disease subgroup were 3.9% (0.24) and 3.9% (0.27). Mean (standard error) annual decline in FVC volume in the more advanced disease subgroup was 146.1 mL (15.5) and 137.6 mL (16.7) following previous pirfenidone and placebo treatment, respectively. Corresponding values in the less advanced disease subgroup were 151.7 mL (10.0) and 156.0 mL (11.2).

Overall, 187 (100.0%) patients with more advanced and 408 (99.8%) patients with less advanced disease experienced ≥1 AE during 180 weeks of treatment (Table 1). AE incidence per patient-year exposure was 8.29 and 7.40 in the more and less advanced disease subgroups, respectively. Both subgroups exhibited a similar pattern of AEs; however, AEs related to IPF progression were experienced by a higher proportion of patients with more advanced versus less advanced disease (56.1% vs 39.6% for dyspnoea; 58.3% vs 27.4% for worsening of IPF; Table 1). Nausea and diarrhoea were each experienced by a higher proportion of patients with less dvanced than more advanced disease (37.7% vs 29.9% for nausea; 30.1% vs 23.5% for diarrhoea; Table 1); however, there were similar AE incidences per patient-year exposure in the more and less advanced disease subgroups (0.19 vs 0.18 for nausea; 0.19 vs 0.16 for diarrhoea). Discontinuation rates due to any reason, AEs related to IPF progression and deaths were each higher in the more advanced than less advanced disease subgroup (Table 1). Throughout the treatment period there was little difference in mean body weight of patients with more versus less advanced disease (baseline: 85.8 kg vs 85.5 kg; Week 180: 81.2 kg vs 81.8 kg).

**Table 1:** Summary of common adverse events^a^ and reasons for treatment discontinuation by IPF severity at RECAP baseline

	More advanced disease (*n* = 187)	Less advanced disease (*n* = 409)	Total (*n* = 596)
Number of patients with ≥1 adverse event in the first 180 weeks of treatment, n (%)			
Total	187 (100.0)	408 (99.8)	595 (99.8)
Cough	96 (51.3)	209 (51.1)	305 (51.2)
Dyspnoea	105 (56.1)	162 (39.6)	267 (44.8)
Fatigue	68 (36.4)	164 (40.1)	232 (38.9)
Worsening of IPF^b^	109 (58.3)	112 (27.4)	221 (37.1)
Nausea	56 (29.9)	154 (37.7)	210 (35.2)
Upper respiratory tract infection	61 (32.6)	137 (33.5)	198 (33.2)
Bronchitis	51 (27.3)	130 (31.8)	181 (30.4)
Diarrhoea	44 (23.5)	123 (30.1)	167 (28.0)
Nasopharyngitis	40 (21.4)	117 (28.6)	157 (26.3)
Dizziness	39 (20.9)	105 (25.7)	144 (24.2)
Headache	37 (19.8)	98 (24.0)	135 (22.7)
Back pain	36 (19.3)	91 (22.2)	127 (21.3)
Dyspepsia	26 (13.9)	96 (23.5)	122 (20.5)
Reasons for discontinuation, n (%)			
All reasons	134 (71.7)	177 (43.3)	311 (52.2)
Adverse event	81 (43.3)	110 (26.9)	191 (32.0)
Related to IPF^c^	26 (13.9)	21 (5.1)	47 (7.9)
Not related to IPF	55 (29.4)	89 (21.8)	144 (24.2)
Withdrawal by patient	16 (8.6)	35 (8.6)	51 (8.6)
Death	20 (10.7)	13 (3.2)	33 (5.5)
Lung transplantation	12 (6.4)	14 (3.4)	26 (4.4)
Physician decision	5 (2.7)	3 (0.7)	8 (1.3)
Other	0 (0.0)	2 (0.5)	2 (0.3)

*IPF* Idiopathic pulmonary fibrosis

^a^In ≥20% of total patients

^b^Worsening of the underlying disease from baseline

^c^Adverse event designated with the preferred terms ‘IPF’, ‘disease progression’ or ‘interstitial lung disease’

## Conclusion

These post-hoc analyses of RECAP found that annual rate of FVC decline was similar with longer-term pirfenidone treatment in patients with more and less advanced IPF (3.4–3.9%), and in line with that of the pirfenidone arm at 52 weeks in CAPACITY (~ 5%) [6]. Moreover, the safety profile of pirfenidone was generally similar between patients with more and less advanced disease (except for AEs related to IPF progression) and in line with that of pirfenidone in ASCEND and CAPACITY [6, 7]. However, the discontinuation rate, particularly due to AEs related to IPF, was higher for patients with more advanced than less advanced disease, which could reflect the higher severity of IPF in the more advanced versus less advanced disease subgroup.

Limitations of this study include the high discontinuation rate, the relatively small number of patients with more advanced disease, and that these were post-hoc exploratory analyses of an open-label extension study without a placebo arm. Additionally, the study population was biased towards patients who had completed CAPACITY, with patients in the previous pirfenidone subgroup having tolerated pirfenidone treatment for ≥72 weeks prior to being categorised by severity of IPF.

In summary, longer-term pirfenidone treatment resulted in a similar rate of lung function decline and safety profile in patients with more advanced versus less advanced IPF. These data suggest that pirfenidone is efficacious, well tolerated and a feasible treatment option in patients with more advanced IPF.

## Abbreviations

AE: Adverse event
DLco: Carbon monoxide diffusing capacity
FVC: Forced vital capacity
IPF: Idiopathic pulmonary fibrosis
SD: Standard deviation
